# Modulation of Cellular Redox Parameters for Improving Therapeutic Responses in Multiple Myeloma

**DOI:** 10.3390/antiox11030455

**Published:** 2022-02-25

**Authors:** Alessandro Allegra, Claudia Petrarca, Mario Di Gioacchino, Marco Casciaro, Caterina Musolino, Sebastiano Gangemi

**Affiliations:** 1 Division of Hematology, Department of Human Pathology in Adulthood and Childhood “Gaetano Barresi”, University of Messina, 98125 Messina, Italy; cmusolino@unime.it; 2 Center for Advanced Studies and Technology, G. D’Annunzio University, 66100 Chieti, Italy; claudia.petrarca@unich.it; 3 Institute for Clinical Immunotherapy and Advanced Biological Treatments, 65100 Pescara, Italy; 4 Unit and School of Allergy and Clinical Immunology, Department of Clinical and Experimental Medicine, University of Messina, 98125 Messina, Italy; marco.casciaro@unime.it (M.C.); gangemis@unime.it (S.G.)

**Keywords:** multiple myeloma, oxidative stress, endoplasmic reticulum stress, reactive oxygen species, chemoresistance, proteasome inhibitors, immunomodulatory drugs, autologous stem cell transplantation

## Abstract

Raised oxidative stress and abnormal redox status are typical features of multiple myeloma cells, and the identification of the intimate mechanisms that regulate the relationships between neoplastic cells and redox homeostasis may reveal possible new anti-myeloma therapeutic targets to increase the effectiveness of anti-myeloma drugs synergistically or to eradicate drug-resistant clones while reducing toxicity toward normal cells. An alteration of the oxidative state is not only responsible for the onset of multiple myeloma and its progression, but it also appears essential for the therapeutic response and for developing any chemoresistance. Our review aimed to evaluate the literature’s current data on the effects of oxidative stress on the response to drugs generally employed in the therapy of multiple myeloma, such as proteasome inhibitors, immunomodulators, and autologous transplantation. In the second part of the review, we analyzed the possibility of using other substances, often of natural origin, to modulate the oxidative stress to interfere with the progression of myelomatous disease.

## 1. Introduction

### 1.1. General Consideration on Multiple Myeloma and Oxidative Stress

Despite recent improvements in the treatment of multiple myeloma (MM) due to the discovery of novel drugs such as proteasome inhibitors, immunomodulatory agents, and monoclonal antibodies, MM remains an incurable tumor with a 5-year percentage of about 54% [1]. Furthermore, myeloma patients undergo relapses that necessitate further treatments, underlining the imperative necessity to recognize the mechanistic drivers of chemoresistance to attain better results.

In healthy cells, redox equilibrium is conserved by sophisticated control of reactive oxygen species (ROS) production and antioxidant defense systems. The damaging consequences of ROS are compensated by a complex system including enzymatic antioxidants such as glutathione peroxidase (GPX), superoxide dismutase (SOD), catalase, thioredoxin reductase, and peroxiredoxin, and non-enzymatic antioxidants such as vitamin C, glutathione (GSH), thioredoxin, vitamin E, and metallothionein, and an imbalance of the oxidative system appears to cause aging and the onset of numerous pathologies [2,3].

### 1.2. Oxidative Stress and Cancer

An augmented concentration of ROS has long been involved both in cancer onset and tumor progression, acting on cellular growth and chemoresistance. Nevertheless, extreme ROS generation or compromised ROS scavenging capacity can provoke cellular damage and death [4]. Several elements can participate in augmenting oxidative stress, and different causes of ROS have been recognized. For instance, mitochondria are essential components of ROS produced from complex I [5], and mitochondrial ROS have acquired consideration for playing a critical role in tumorigenesis. Similarly, endoplasmic reticulum (ER) oxidative protein folding is a different source of ROS. Much data proposes a strong correlation between oxidative stress and ER stress, confirmed by common signaling pathways. Alterations of intracellular equilibrium can provoke a disturbed regulation of cellular activities such as programmed cell death [6].

Protracted oxidative stress provokes damage in DNA; the most common base altered is guanine in DNA and forms 8-hydroxydeoxyguanosine (8-OHdG), which can join to thymidine rather than cytosine. The amount of 8-OHdG is commonly considered a deleterious DNA alteration and is responsible for the mutagenesis [7]. Oxidative DNA damage can provoke the loss of epigenetic information, generally due to the damage in CpG island methylation assets in promotor regions of a gene [8]. Therefore, considering all these factors, it suggests that oxidative stress may cause several diseases, including cancer.

A strong relationship has also been reported between the oxidative status of patients with multiple myeloma and the onset and progression of the disease. Secretory cells like plasma cells have been valued for generating 3–6 million disulfide bonds per minute, which provokes the generation of an equivalent quantity of intracellular ROS [9], which can operate as a second messenger influencing MM beginning and MM progression [10].

The correlation between MM progression and the diminution of antioxidants and the augment of pro-oxidant substances in MM patient serum has been reported in numerous studies. Assessed serum concentrations of antioxidants such as vitamin C and vitamin E, SOD1, GPX, and catalase were reduced in patients compared to healthy subjects. In contrast, serum number of oxidative markers such as advanced oxidation protein products, malondialdehyde (MDA) and other markers of fatty acid peroxidation were more significant [11,12,13,14,15]. Analogously, levels of antioxidants capable of preventing lipid peroxidation, such as paraoxonase and arylesterase, were reduced in MM patient sera [16,17].

In a previous study, we determined that sirtuin2 and sirtuin3, molecules implicated in numerous cell signaling pathways, are reduced in MM and that minor levels were associated with an advanced stage of disease and redox imbalance [18]. In the peripheral blood mononuclear cells of these patients, we found a disproportion of oxidative stress markers with trim levels of the antioxidant enzyme GPX, reduced quantities of nicotinamide adenine dinucleotide (NAD) + and more significant amounts of pro-oxidant enzyme hydrogen peroxide (HP) with respect to healthy subjects (Table 1).

### 1.3. Oxidative Stress and Myelomagenesis

There are numerous systems through which a modified oxidative equilibrium can provoke the onset of MM, such as the stimulation of genetic mutations and a modification of angiogenic dynamics. Oncogenes such as MYC cause DNA injury in MM cells by augmented replicative stress and increased oxidative stress [19]. It has been suggested that altered regulation of one CCND gene coding cyclin D is an essential phenomenon of MM pathogenesis [20] due to its cell cycle controlling capacity [21]. Cyclin D1 expression causes an alteration of the redox equilibrium by generating ROS. The consequential oxidative stress stimulates the p44/42 mitogen-activated protein kinase (or ERK1/2) signaling pathway, augmented cell adhesion to fibronectin, and modified the sensitivity to different drugs [22].

**Figure 1:**
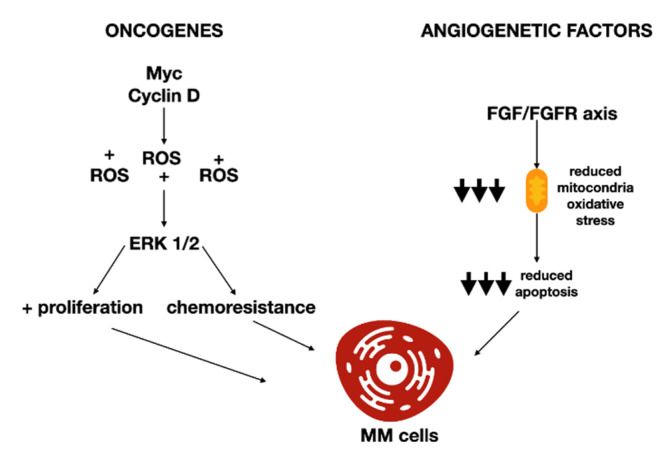
Oncogenes and angiogenetic factors’ oxidative stress interplay in MM.

However, numerous other factors and pathways related to oxidative stress seem capable of intervening in myelomagenesis. Fibroblast growth factors (FGF) operate as proangiogenic factors in MM. A study established that the FGF/FGFR axis is crucial for MM cell viability and proliferation by defending MM cells from oxidative stress-caused programmed cell death [23]. FGF/FGFR reduced the proliferation of MM cells by stimulating mitochondrial oxidative activity while the antioxidant vitamin E or mitochondrial catalase overexpression reduced the programmed cell death (Figure 1). Moreover, mitochondrial oxidative stress arose due to proteasomal degradation of the c-Myc oncoprotein that caused glutathione depletion. Correspondingly, the production of a proteasome non-degradable c-Myc protein mutant was adequate to prevent glutathione reduction and rescue the stimulus of programmed cell death due to FGF blockade. These results were validated on bortezomib-resistant MM cell lines and on primary MM cells from the bone marrow of MM subjects.

However, a change of the oxidative condition is not only responsible for the onset of MM and its progression, but it also seems fundamental for the therapeutic response and the emergence of any drug resistance [24,25]. Our review aimed to analyze the existing literature data on the role of oxidative stress in the response to drugs commonly used in the treatment of MM, such as proteasome inhibitors, immunomodulators, and autologous transplantation. In the second part of the review, we analyzed the possibility of using other substances, often of natural origin, to modulate oxidative stress to interfere with the progression of myelomatous disease.

## 2. Oxidative Stress and Proteasome Inhibitors

The employment of the proteasome inhibitor bortezomib (BTZ) has radically modified the survival of MM subjects when utilized as a single agent or in combination with other drugs such as dexamethasone. Recently, other proteasome inhibitors have been FDA- and EMA-approved for therapy of MM patients comprising those refractory to BTZ administration such as carfilzomib (CFZ) and ixazomib (IXZ) [26,27].

Proteasome inhibition causes an increase in unfolded proteins, which activate ER stress [28,29]. This stress is believed to be the main intermediary of the toxicity of proteasome inhibitors, causing cell death through different systems comprising an augmented generation of ROS [28,29]. Oxidative stress has been recognized as an important method of BTZ action in myeloma cells [30,31,32]. For example, ROS production comes first at the start of the BTZ-provoked programmed cell death process [33,34]. Furthermore, co-administration of the antioxidant tiron prevented BTZ-caused ROS production and cytotoxicity [35,36].

A group of researchers has recognized a diverse mechanism of BTZ- and CFZ-dependent generation of oxidative stress in MM cells. They identified the transcription regulator Kruppel-like Factor 9 (KLF9) as an intermediary of the effects of BTZ [37]. BTZ administration augmented KLF9 in cultured MM cells [37]. In contrast, KLF9 raised ROS concentrations in normal fibroblasts through transcriptional repression of numerous genes involved in ROS metabolism, comprising the gene for TXNRD2 [38], which is essential for ROS scavenging in mitochondria [39,40]. The protein coded by the TXNRD2 gene belongs to the pyridine nucleotide-disulfide oxidoreductase family and is a component of the thioredoxin system. It functions as a homodimer containing FAD and selenocysteine (Sec) at the active site. Sec is coded by a UGA codon that usually signals translation termination. The 3′ UTRs of selenoprotein mRNAs contain a conserved stem-loop structure, the Sec insertion sequence element, which is necessary to recognize UGA as a Sec codon rather than as a stop signal. Alternatively, spliced transcript variants encoding different isoforms, comprising a few localized in the cytosol and some lacking the C-terminal Sec residue, have been reported for this gene. It controls reactive oxygen species levels and regulates redox homeostasis, maintains thioredoxin in a reduced state, and may play a role in redox-regulated cell signaling.

Fink et al. described a similar mechanism by which BTZ and CFZ can provoke oxidative and ER stresses in MM cells via an effect on mitochondrial TXNRD2 [41]. Reduction in TXNRD2 to the amounts discovered in BTZ- or CFZ-treated cells induces ER and oxidative stress and cell death similar to those provoked by proteasome inhibitors. Similarly, the reestablishment of regular TXNRD2 quantity in MM cells after administration of proteasome inhibitors decreases ER, oxidative stress, and cell death, and ectopic production of TXNRD2 in MM cell xenografts in immunocompromised animals reduces the efficacy of BTZ [41].

However, other pathways can be called into question to justify the oxidative stress-related anti-myeloma action of the proteasome inhibitors. Peroxisome proliferator activated receptor-γ coactivator-1α (PGC-1α) is an activator that controls the genes involved in mitochondrial genesis and energy metabolism [42]. Still, it is also the primary controller of external stimuli such as ROS or other signals [43,44,45,46,47]. Numerous experiments evaluated the function of PGC-1α in malignancies [48]. The findings of these studies are surprising as PGC-1α has a different effect on cancer progression by stimulating tumor cell vitality after cellular stress and augmenting the programmed cell death via the control of Bcl-2 and Bax expression. In summary, PGC-1α is increased in MM and PGC-1α modifies angiogenesis by controlling Vascular Endothelium Growth Factor.

Consequently, PGC-1α appears to play a pro-cancer role in MM, and PGC-1α expression could be important in the effectiveness of the treatment. An in vitro study evaluated the expression of PGC-1α, catalase (CAT), and SOD-2 after administration of BTZ or dexamethasone. After chemotherapy, the expression of PGC-1α and SOD-2 was augmented but associated with a boost of ROS. After the reduction in PGC-1α, ROS amounts and the programmed cell death induced by BTZ were increased. These data proposed that PGC-1α modifies ROS in MM and that suppression of augmented PGC-1α after chemotherapy causes a further augment of ROS by reducing the antioxidant component, finally increasing the antineoplastic action of BTZ [49].

The importance of oxidative stress modification in the therapeutic efficacy of proteasome inhibitors has been confirmed in other studies where the administration of CFZ and LBH589 (panobinostat) caused a conspicuous increase in ROS in MM cells, and NAC reduced oxidative stress, as well as the consequent programmed cell death [50].

In conclusion, plasma cells are programmed to generate immunoglobulins and can fabricate thousands of immunoglobulins per second. This specific secretory faculty is extraordinary; however, it happens at a cost to the cell. A significant amount of protein production is physiologically demanding, and plasma cells are distinguished by augmented quantities of cellular stress that comprise high degrees of oxidative stress, as protein disulfide bond generation produces equimolar amounts of ROS. Thus, the peculiar biology of the plasma cells makes MM distinctively susceptible to alterations in oxidative stress homeostasis, a sensitivity that can be utilized therapeutically.

### Oxidative Stress and Chemoresistance to Proteasome Inhibitors

MM cells attain chemoresistance to proteasome inhibitors, actuating protection systems, such as decreasing oxidative stress, reducing proapoptotic signals, and augmenting anti-apoptotic pathways. Understanding the modalities via which MM cells develop resistance to anti-MM drugs has identified several substances that can reestablish the therapeutic answer by provoking MM cell death.

A changed redox control influences BTZ resistance in MM. MM cell lines resistant to BTZ maintain high oxidative stress and are cross-resistant to ER stress-causing elements tunicamycin and thapsigargin. Furthermore, cells presenting elevated/wild type concentrations of glutathione S-transferase P (GSTP) are more resistant than GSTP1/P2 knockout cells. Similarly, concentrations of S-glutathionylated proteins are augmented in resistant cells. GSTP mediated S-glutathionylation (SSG) controls the effects of several redox functional ER proteins. A study stated that post-translational change regulates the equilibrium between foldase and ATPase performances of the binding immunoglobulin protein (BiP). BiP generation and S-glutathionylation are augmented in the bone marrow from MM subjects versus healthy subjects. Inhibiting SSG in MM cells with a GSTP inhibitor reestablished BiP functions and annulled resistance to BTZ [51].

A proteomic analysis performed on plasma cells of MM patients treated with BTZ, doxorubicin and dexamethasone (PAD) discovered 118 proteins (35 augmented and 83 decreased) [52]. A group of these proteins comprised proteins implicated in the reaction of oxidative stress, comprising peroxiredoxins, and TXNDC5, which operate as antioxidants, and these proteins were augmented in BTZ-resistant subjects.

Other reports propose that inhibiting proteasomal function at the level of proteasome-associated deubiquitinases (DUBs) offers a system to overwhelm BTZ resistance. The molecule b-AP15 is a substance able to block the proteasome-associated DUB action that provokes an increase in ROS amounts in tumor cells. Antioxidants reduce programmed cell death induction by b-AP15. Oxidative stress induction by b-AP15 is abolished in cells deprived of mitochondrial DNA. In contrast, a reduced generation of cytochrome c oxidase subunit 5b and translocase of external mitochondrial membrane was demonstrated in b-AP15-treated cells. These data propose a mitochondrial derivation of the augmented amounts of ROS reported in cells treated with b-AP15 [53].

Hemeoxygenase-1 (HO-1) is an enzyme in mammalian cells and is an essential factor in heme catabolism. HO-1 is produced in small quantities, but it is increased after different stimuli such as oxidative stress. This augmentation is a protection system that increases cell survival [54]; in tumor cells, HO-1 is recognized to have a major effect on protection of neoplastic cells against chemotherapy-caused increase in ROS [55].

An experiment was undertaken to study the effect of HO-1 in MM after BTZ administration and determine how HO-1 is involved in the occurrence of BTZ resistance [56]. Data demonstrated that BTZ caused cell death in diverse MM cell lines and provoked ROS generation and ER stress. A relevant increase in HO-1 was also reported, but this outcome was inhibited by parallel administration of 4-phenybutirric acid, a molecular chaperone, which is recognized to decrease ER stress. Unexpectedly, blockage of HO-1 functions with SnMP, a competitive HO inhibitor, was incapable of augmenting BTZ sensitivity in MM cells, while the blockage of HO-1 nuclear translocation by E64d increased sensitivity to BTZ [56]. Therefore, these results propose that BTZ sensitivity is due to HO-1 nuclear localization and not to its enzymatic function. This finding may be a critical instrument to overwhelm BTZ resistance in MM subjects.

A different study evaluated the possibility that Toll-like receptor 4 (TLR4) may be involved in the mechanisms of BTZ resistance [57]. Some authors demonstrated that TLR4 stimulation caused mitochondrial generation and augmented mitochondrial amount in MM cell lines. Furthermore, the TLR4 pathway was stimulated after BTZ administration and was raised in BTZ-resistant U266 cells. The combined administration of BTZ with TAK-242, a specific TLR4 inhibitor, overwhelmed chemoresistance via the production of more significant oxidative stress, mitochondrial depolarization, and critical alteration of mitochondrial functions, which provoked an energy emergency and augmented programmed cell death. These data suggest that TLR4 operates as a system that protects mitochondria after BTZ administration, supporting mitochondrial activities and enhancing chemoresistance. Thus, blockade of TLR4 could represent a valid target in refractory MM patients to restore BTZ sensitivity.

During recent years, other research demonstrated the role of mitochondria in the MM-acquired chemoresistance [58], and numerous anti-mitochondrial substances were identified as molecules able to overwhelm drug resistance. The actual mechanisms of action would appear to consist of caspase-independent pathways, essentially founded on augmented oxidative stress, and among the substances considered, we can mention ascorbic acid, VLX1570, 2-methoxyestradiol, artesunate, dihydroartemisinin, and Erw-ASNase. However, different substances reestablish the effectiveness of proteasome inhibitors via caspase-dependent mechanisms, such as AT-101, SMAC-mimetics, glutaminase-1-inhibitors, KD5170, NOXA inhibitors, FTY720, CDDO-Im, GCS-100, a derivative of ellipticine, and thenoyltrifluoroacetone. These molecules enhanced the effectiveness when used together with the other anti-myeloma drugs used [59,60].

Thus, protection components able to defend from oxidative stress would appear to shelter MM cells from drugs’ effects. Scavenger receptor class A, member 3 (SCARA3), is a component of the scavenger receptor family [61,62]. Some studies recognized that the expression of SCARA3 was induced by oxidative stress, and it is considered a cellular stress response (CSR) gene as it can defend cells from ROS [61].

A study assessed the action of SCARA3 in MM. MM cell lines and SCARA3 expression were stimulated after employing oxidative stressors such as ionizing radiation [63]. Furthermore, in these cell lines, the presence of epigenetic inactivation of SCARA3 was observed. An increased SCARA3 concentration reduced myeloma cell destruction operated by BTZ and dexamethasone and BTZ, while SCARA3 knockdown augmented the sensitivity of MM cells to these drugs. A clinical study demonstrated an inverse correlation between SCARA3 gene expression, myeloma progression, and SCARA3 expression. Finally, SCARA3 could be used as a prognostic factor of therapeutic response in MM patients.

Finally, polymorphisms of genes coding Tumor Necrosis Factor-alpha (TNF-α) and glutathione S-transferase (GSTs) may also modify the prognosis of MM patients. An experiment evaluated the correlation between deletion polymorphisms in GSTT1/GSTM1 genes and single nucleotide polymorphisms in the TNF-α gene at positions −308/−238 with the prognosis in MM patients, while an in vitro study assessed the sensitivity to BTZ. After BTZ administration, results demonstrated a considerably more significant number of apoptotic cells in GSTT1-present, GSTM1-null/present, −308GG and −238GG/GA+AA genotypes [64].

Similar results were found evaluating the onset of chemoresistance against other proteasome inhibitors such as CFZ and IXZ.

Prelowska et al. recognized a robust therapeutic methodology to avoid acquired resistance to proteasome inhibitors. The pleiotropic effects of ʟ-glutamine (Gln) in cellular activities makes alteration of Gln metabolism a possibly valid candidate for combination treatment with CFZ. The authors reported that plasma cells, both sensitive and resistant to PIs, present membrane Gln transporter (ASCT2), need extracellular Gln for survival, and are susceptible to ASCT2 inhibitors (ASCT2i). ASCT2i synergistically augment the cytotoxic efficacy of CFZ by causing programmed cell death and regulating autophagy. Administration of ASCT2 inhibitor V9302 and CFZ increases the intracellular concentrations of ROS and oxidative stress markers and stimulates catastrophic UPR [65].

In a different study, employing human myeloma cell lines and patient-derived CD138+ cells, the authors compared gene expression patterns between innate proteasome inhibitor-sensitive and proteasome inhibitor-resistant myeloma cells following test dosing with the second-generation proteasome inhibitor IXZ. They found 1553 genes that changed after therapy in sensitive cells versus only seven in resistant cells. Ingenuity pathway analysis utilizing top kinetic response genes recognized the mediated oxidative stress response as a top canonical pathway in IXZ-sensitive cell lines and patient-derived cells. Additionally, in this case, modulating the oxidative stress could determine sensitization to the administration of the proteasome inhibitor [66].

In conclusion, there is no doubt that oxidative stress plays a role in the onset of chemoresistance to classical anti-MM drugs. Inducing high ROS amounts in MM cells, provided that high, lethal concentrations are indeed reached, seems to be a productive strategy. Probably the most promising approach relies on the employment of a combination of drugs that can intervene in multiple pathways while reducing mitochondrial stimulation, decreasing antioxidant defenses, and further augmenting ROS production.

## 3. Oxidative Stress and Immunomodulatory and Alkylating Drugs

Immunomodulatory drugs (IMiDs) such as thalidomide, lenalidomide, and pomalidomide represent an essential element for the therapy of MM subjects. These drugs need the presence of cereblon (CRBN), a molecule able to cause specific ubiquitination and destruction of two lymphoid transcription factors, IKZF1 and IKZF3 [67,68].

Much data has permitted us to recognize the essential correlation between IMiDs, CRBN, and the increase in oxidative stress [69]. MM cells with decreased antioxidant systems showed higher sensitivity to lenalidomide-provoked programmed cell death and a report foresaw IMiD sensitivity in MM through the analysis of baseline antioxidative stress capability [70].

**Figure 2:**
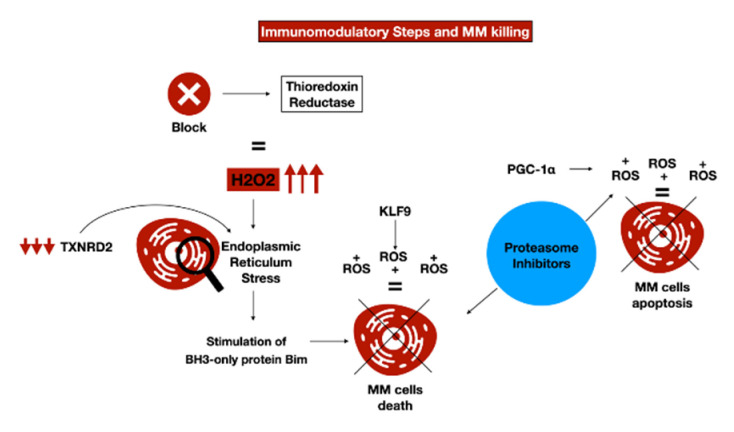
Immunomodulatory steps and MM cell death with the proteasome inhibitors’ potential effects.

IMiDs operate principally by blocking peroxidase-caused intracellular H_2_O_2_ degradation in MM cells. MM cells with lesser H_2_O_2_-degradation ability were more susceptible to lenalidomide-provoked H_2_O_2_ increase and subsequent cellular toxicity. The CRBN-caused metabolization of IKZF1 and IKZF3 was an effect of H_2_O_2_-caused oxidative stress. Lenalidomide augmented the content of H_2_O_2_ by blocking thioredoxin reductase in cells presenting CRBN, producing an increase in immunoglobulin light-chain dimers, causing an extension of ER stress, and provoking cellular damage through stimulation of BH3-only protein Bim in MM cells. Similar effects are produced employing different thioredoxin reductase inhibitors (Figure 2). The authors settled a novel procedure for ascertaining total cellular antioxidative ability, which can be employed to foresee which MM subjects might reap the best benefits from a IMiDs-based treatment [71].

Alkylating substances are commonly employed drugs in the therapy of solid tumors, and hematologic malignancies, including lymphoma and leukemia. Melphalan was the most frequently utilized agent against MM. Nevertheless, despite a 70–80% initial overall response, all treated subjects relapsed due to chemoresistance. By employing transcriptomic and proteomic analysis on melphalan sensitive and resistant cell lines, an experiment recognized modifications in cellular pathways not before correlation with the onset of Melphalan chemoresistance in MM cells, comprising a metabolic change compliant with the Warburg effect and augmented oxidative stress response due to the signaling of vascular endothelial growth factor/IL8 [72]. Finally, specific oxidative stress response enzymes were targeted by inhibitors, some of which showed cytotoxic activity against the melphalan-resistant cells and could be investigated to clarify their possible ability to overwhelm melphalan resistance [72].

## 4. Oxidative Stress and Autologous Transplantation in Multiple Myeloma

Some studies have shown that the high-dosage treatment employed for the conditioning regimen for autologous stem cell transplantation (HSCT) is related to the augmented generation of ROS and reduction in several elements of the antioxidant system [73]. According to the fact that ROS influences the production of different growth factors that can modify cell cycle progression in numerous types of cells, including hematopoietic progenitors, oxidative stress has been recognized as a fundamental system to elucidate the appearance of tissue injury after HSCT [74,75].

A study examined the correlation between oxidative stress and DNA injury with grafting time in MM subjects after HSCT [76]. Markers of oxidative stress and DNA damage index (DI) were studied before HSCT (pre-CR), during the conditioning regimen (CR), and after HSCT (up to 20 days after HSCT). Results demonstrated that CR MM subjects had an hyperoxidative state with high DI with respect to pre-CR and to the control group. Nevertheless, oxidative stress was reduced in the following period returning to baseline values. MM subjects displayed a negative correlation between the grafting time and basal amounts of GPX, suggesting that lesser quantities of GPX are connected to a lengthier grafting time.

In contrast, for the DI, the correlation was positive. These results suggest the potential of these markers as prognosticators of grafting time and tissue damage in MM subjects submitted to HSCT [76], and these results were confirmed by other studies [77]. These data could be of extreme importance as the longer the grafting time, the greater the possibility of complications such as infections that can provoke death [78].

On these results, an attempt was undertaken to change the oxidative stress in these subjects to decrease cellular and tissue damage. The nonsteroidal anti-inflammatory substance meloxicam mobilizes blood stem cells and can reduce mobilization-caused oxidative stress on stem cells. In an experiment, the authors assessed if meloxicam administration before filgrastim dispensation augments collection of CD34^+^ cells after HSCT [79]. Data showed a substantial reduction in oxidative phosphorylation, probably due to SIRT1, indicating that meloxicam may neutralize oxidative stress after HSCT. As meloxicam is a low-cost and safe complement, it might be an easy method for improving graft quality.

## 5. Future Perspectives on the Modulation of Oxidative Stress in Patients with Multiple Myeloma

Apart from arsenic trioxide, which has exhibited inadequate effectiveness [80], no other substances that can cause increased oxidative stress are currently being employed in the MM treatment. Nevertheless, in vitro studies and preclinical experimentation findings indicate that stimulation of oxidative stress is an efficient MM treatment approach, and the combined administration of BTZ and several ROS-stimulating molecules showed promising results. For instance, the addition of an inhibitor of thioredoxin 1 PX-12 (1-methylpropyl 2-imidazolyl disulfide) or auranofin, a substance able to inhibit thioredoxin reductases 1 and 2 [81] in MM cell cultures, induces a ROS-dependent increase in MM-programmed cell death [82]. Analogous findings were achieved by genetic inhibition of TRX1 or TXNRD1, as a study stated that the reduction in TXNRD2 through shRNA causes apoptosis in MM cells. In an in vivo study, auranofin decreased tumor mass and augmented survival in a transgenic mouse model of chronic lymphocytic leukemia [83]. These results indicate that auranofin could be employed for the therapy of MM both alone and along with proteasome inhibitors (Figure 2).

Several other substances able to cause increased oxidative stress and cytotoxicity in MM cells have been recognized in recent years. These molecules comprise b-AP15, a small molecule able to inhibit the ubiquitin peptidase that also has anti-TXR and anti-TXNRD capacities; cis-bixin able to inhibit TXR and TXNRD1; motexafin gadolinium, a substance that blocks TXNRD and ribonucleotide reductase; chaetocin, a factor inhibiting histone methyltransferases; parthenolide, a substance with anti-inflammatory activity; imexon (Amplimexon/NSC-714597), a cyanoaziridine molecule that stimulates oxidative stress with an unidentified mechanism [84,85,86,87,88,89,90,91,92].

A different attempt was performed by studying the mucin 1 C-terminal subunit (MUC1-C), an oncoprotein abnormally expressed in MM cells. Considering MUC1-C with GO-203, an inhibitor of MUC1-C homodimerization, it was possible to cause ROS-mediated MM cell death. Moreover, GO-203 and BTZ synergistically reduced production of the p53-inducible regulator of glycolysis and apoptosis (TIGAR), which stimulated shunting of glucose-6-phosphate into the pentose phosphate pathway to produce reduced GSH [93]. The study also stated that GO-203 is effective against BTZ-resistant MM cells, as BTZ resistance is due to BTZ-caused increases in GSH and TIGAR concentrations and GO-203 resensitizes BTZ-resistant cells to BTZ therapy by reducing GSH and TIGAR [93].

A diverse approach could be represented by interfering with the ionic delivery and its action on the oxidative equilibrium. Transient Receptor Potential Vanilloid type 1 (TRPV1) is the best-known component of the vanilloid receptor subfamily of ion channels [94].

TRPV1 was present in MM cell lines and primary MM cells. The use of the antagonist AMG9810, a substance able to inhibit TRPV1, could provoke MM-programmed cell death and operate synergistically with BTZ, overwhelming chemoresistance [95]. Moreover, the ability to inhibit TRPV1 increased mitochondrial calcium concentrations with consequent mitochondrial ROS increases and depolarization. These results were upturned by the administration of calcium chelators, proposing an effect of calcium changes on oxidative stress. Furthermore, AMG9810 inhibited BTZ-caused increase in mitochondrial HSP70 and reduced protective mitochondrial unfolded protein response. The proteomic analysis demonstrated a specific molecular signature that correlated with the change of the ubiquitin signaling pathway, with 38 proteins reduced upon combined bortezomib/AMG9810 [95].

Other attempts to interfere with oxidative stress in MM have been conducted via the employment of substances able to change ER stress, of molecules capable of operating at the same time on the antioxidant systems and ubiquitin–proteasome system, via the employment of prostaglandins or other molecules capable of modifying the antioxidative effects performed by the nuclear factor erythroid2 like 2 (NFE2L2, also known as NRF2) or acting on the p53 pathway.

For instance, protein disulfide isomerases (PDIs) are a group of more than 20 ER oxidoreductase enzymes [96]. They act by generating disulfide bonds between cysteine residues, and their catalytic activity is redox dependent. Robinson et al. recognized several PDI members as the molecular targets of E61 [97]. PDIs control oxidative protein folding and E61 administration stimulated ER and oxidative stress and the increase in ubiquitinylated proteins. In an experimental animal model, administration of a new form of PDIs inhibitor, E64FC26, enhanced survival and augmented the efficacy of BTZ without any collateral effects.

Anchoori et al. designed ubiquitin–proteasome inhibitors aiming to interfere with the action of ubiquitin receptor RPN13 within the proteasome’s 19S particle [98]. Administration of RA375 provoked a fast and strong increase in polyubiquitinated proteins and decreased intracellular GSH concentrations, which caused ER and oxidative stress and stimulated programmed cell death in MM cell lines [98].

15-DeoxyΔ12,14-prostaglandin J2 (15d-PGJ2) is a ligand of PPAR-γ with antiproliferative, anti-inflammatory, and proapoptotic functions [99]. Inhibition of PPAR-γ by 15d-PGJ2 reduces angiogenesis and causes programmed cell death in tumor cell lines. However, the most interesting aspect for our discussion is that ROS production has been described 2 h after administration of 15d-PGJ2 and numerous elements of the protein kinase B (AKT) and protein kinase A-Polo-like kinase 1 (PKA-PLK1) pathways are modified by ROS production, causing programmed cell death through a reduction in AKT and PLK1 [100].

A different study assessed the antiproliferative action of 15dPGJ2 on MM in vitro and in vivo via ER and oxidative stress pathways [101]. 15d-PGJ2 decreased cell growth augmented programmed cell death, and these effects were due to an increase in stress-related genes and an increase in oxidized glutathione concentrations. Furthermore, 15d-PGJ2 at 4 mg/kg in vivo arrested MM growth.

As mentioned above, NRF2 influences MM cells, but it has various actions in normal and neoplastic cells [102]. Oxidative stress provokes the generation of Nrf2 from Keap1 and subsequent nuclear translocation of NRF2, where it connects to the antioxidant response element (ARE). This molecule controls cytoprotective genes implicated in oxidative stress response [103]. Reduction in NRF2 function provokes the augmentation of harmful ROS concentrations in MM cells distinguished by a high ROS turnover while saving normal cells where basal ROS production is marginal. Only a few substances that inhibit NRF2 have been described comprising brusatol [104]. A study reported that such molecules stimulated augmented oxidative stress altering growth and survival of MM cells [105].

Finally, CP-31398 (CP) is a small molecule that stimulates wild-type p53 functions; this causes programmed cell death and cell cycle arrest in MM cell lines and primary MM cells, while it also reduces the growth of MM xenografts in mice [106,107]. However, CP-caused programmed cell death happens irrespective of the p53 condition, indicating that CP has further, unknown mechanisms of action [108].

### 5.1. Use of Substances of Natural Origin in the Modulation of Oxidative Stress in Multiple Myeloma

A previous study demonstrated that combined administration of curcumin with CFZ could cause more decisive cytotoxic action on in vitro cultured U266 cells [109]. U266 cells contact with curcumin or CFZ-augmented ROS concentrations, although their generation did not seem increased after combined administration. Remarkably, NF-κB nuclear increase was diminished by using CFZ or curcumin and was more profoundly reduced in cells exposed to both drugs, probably due to the diverse pathways that affect NF-κB. Furthermore, CFZ and curcumin stimulated the p53/p21 axis and G0/G1 cell cycle arrest, and curcumin augmented the CFZ proapoptotic effect [109].

However, numerous other substances of natural origin can modulate oxidative stress.

Sanguinarine (SNG) is a benzophenanthridine alkaloid extracted from the root of *Sanguinaria canadensis*, which demonstrated antitumor effects in in vivo and in vitro experimentation. Surprisingly, SNG does not display toxic effects in healthy cells. A reduction of more than 70% of neoplastic proliferation has been reported, and it is due to a SNG-mediated generation of ROS and depletion of GSH. A study assessed the possible antiproliferative effects of SNG in MM cell lines such as IM9, U266, MM1S, and RPMI-8226 [110]. SNG use caused a dose-dependent reduction in cell survival via mitochondrial membrane potential loss and stimulation of programmed cell death via stimulation of caspase 3, 9, and cleavage of PARP; the employment of Z-VAd-FMK, a universal caspase inhibitor, reduced MM apoptosis. Furthermore, programmed cell death also occurred for inhibition of the STAT3 pathway. Finally, subtoxic amounts of SNG could augment the cytotoxic effects of BTZ [110].

*Salvia miltiorrhiza* (SM) is also a traditional medicinal herb, and it has been employed for several clinical uses [111], comprising anti-tumor effects in hematological malignancies [112,113]. In a study, SM use dose-dependently reduced the survival of U266 and U937 cells and augmented ROS production. The anti-growth action of SM was abolished by employing the ROS scavenger NAC. Additionally, SM administration boosted ER stress by increasing several factors such as phosphorylated eukaryotic Initiation Factor 2, phosphorylated activating transcription factor 4, and phosphorylated protein kinase RNA-like endoplasmic reticulum kinase. Furthermore, SM augmented the tumor suppressor, miRNA-216b, and inhibited its target, c-Jun, while a miRNA-216b inhibitor reduced the programmed cell death induced by SM [114].

A different substance able to modify the oxidative stress in MM cells is the Caffeic acid phenethyl ester (CAPE), a phenolic molecule found in bee glue. CAPE has anticancer effects, and a study evaluated the anti-myeloma action of CAPE, showing that CAPE decreased the proliferation of MM cells without changing the survival of non-tumoral peripheral blood B cells [115]. CAPE stimulated oxidative stress-response genes comprising heme oxigenase-1. Interestingly, the intracellular ROS amounts were not augmented by CAPE, but the effects encountered after the use of the antioxidant NAC and a glutathione synthesis inhibitor such as buthionine sulfoximine indicated that CAPE might provoke oxidative stress by reducing intracellular antioxidants rather than causing increased ROS levels.

A natural compound, resveratrol (RSV), showed anti-growth effects in MM cell lines [116]. More significantly, a small amount of RSV was synergistic with a small dosage of CFZ to cause programmed cell death in MM cells. Other experimentation demonstrated that mitochondria had a primary role in RSV/CFZ combination treatment. RSV caused the delivery of the second mitochondria-derived activator of caspase (Smac) and maintained the Smac in an elevated concentration after combined administration with CFZ. Furthermore, RSV had a synergistic effect with CFZ to augment ROS generation [116].

Formononetin (FT) is a natural substance, an isoflavone extracted from several plants such as *Pueraria lobata*, *Trifolium pratense*, *Astragalus membranaceus*, and *Glycyrrhiza glabra*. It seems active against MM cell lines and MM xenograft tumors in experimental animal models via the negative control of STAT3 and STAT5 pathways. This effect is mediated by reducing several kinases such as c-Src, JAK1, and JAK2, due to an increased generation of ROS for a GSH/GSSG disequilibrium [117]. Furthermore, FT caused cell cycle arrest, decreased the generation of proliferative, anti-apoptotic, and angiogenetic signals, while in vivo, intraperitoneal FT considerably reduced the MM progression in a MM xenograft mouse model [117].

Plitidepsin, a cyclic depsipeptide extracted from the marine tunicate Aplidium albicans, has been authorized by Australian regulatory authorities to treat MM subjects. Plitidepsin causes oxidative stress and JNK1 phosphorylation, stimulating programmed cell death in MM cells. Furthermore, plitidepsin-triggered ER stress caused unfolded protein response, comprising the alternative splicing of XBP1, and the phosphorylation of eIF2α and JNK [118].

Finally, the natural substance curcusone D, a diterpene extracted from the herbal plant *Jatropha curcas*, was recognized as a new ubiquitin–proteasome pathway (UPP) inhibitor. Curcusone D inhibits cell proliferation and programmed cell death in MM cells, inducing ROS generation, strongly correlated with DUB inhibition that NAC could block. Finally, curcusone D and BTZ displayed intense synergistic action against MM cells [119] (Table 2).

### 5.2. Metabolic Modifications and Oxidative Stress in Multiple Myeloma

The studies that try to modify the oxidative balance through action on metabolism are fascinating. For instance, the metabolism of cholesterol has a role in cancer progression and chemoresistance [120]. Amongst cholesterol metabolites, oxysterols are generated via enzymatic reactions and by auto-oxidation and modify cell growth and induce several forms of cell death comprising oxiapoptophagy in numerous malignancies [121,122,123].

A study assessed the possible anti-myeloma effects of two cholesterol metabolites such as the 5,6 α- and 5,6 β-epoxycholesterol (EC) isomers [124]. These molecules displayed a robust antiproliferative effect in MM cell lines and ex vivo in MM patients’ CD138+ malignant plasma cells. Furthermore, the report confirmed that 5,6 α-EC and 5,6 β-EC provoked oxiapoptophagy via oxidative stress, programmed cell death, and autophagy. Remarkably, the combined administration of the two substances displayed a synergistic effect on MM cells.

Analogously, other studies have suggested the effectiveness of administering high-dose statins in reducing MM cell vitality via interference with cholesterol synthesis. MEDICA analogues are long-chain, α,ω-dioic acids, which can inhibit mitochondrial complex I of the electron transport chain, causing ROS generation [125,126,127,128,129]. In a study, a MEDICA analogue could reduce the survival of MM cells in vitro and suppress the proliferation of MM xenograft in vivo. Inhibition of MM cell growth by MEDICA is correlated with the suppression of the mTORC1, STAT3, and MAPK pathways owed to mitochondrial oxidative stress, which is nullified by adding exogenous cholesterol. Remarkably, it is known that the reduction in MM cell survival induced by BTZ-caused oxidative stress is annulled by adding cholesterol, and the time-to-best-response of MM patients to BTZ administration is positively correlated with plasma cholesterol levels [130].

Finally, a different therapeutic approach was to change the glycolytic and oxidative status. In a study, the authors modified cellular antioxidant capabilities and augmented pro-oxidant activities to cause oxidative stress-mediated toxicity in the MM stem-like population. To inhibit glycolytic and oxidative metabolism, they employed 2-deoxyglucose (2-DG), which has been utilized in clinical trials as an adjuvant to tumor treatment. Hexokinase transforms 2-DG to 2-deoxy-glucose-6-phosphate, a substance that cannot be metabolized by phosphoglucose Isomerase, inhibiting glycolysis and stimulating oxidative stress [131]. Moreover, 2-DG can modify the N-linked protein glycosylation and promote ER stress and programmed cell death. Administration of mitochondrial-targeting agent decyl-triphenylphosphonium (10-TPP) augmented the intracellular pro-oxidant concentration in stem-like and mature MM cells, and the combination of 2-DG with 10-TPP reduced survival of MM cells [131].

## 6. Modulation of Oxidative Stress to Prevent Treatment Side Effects

The research must evaluate the two sides of the coin. On the one hand, the modulation of oxidative stress and its increase can be helpful to increase the efficacy of anti-myeloma drugs. On the other, reducing stress can decrease the toxicity of the treatment and reduce the incidence of some side effects induced by the therapy, such as nephropathy and cardiotoxicity.

Several reports have demonstrated that CFZ administration can induce nephropathy [132].

Rutin, also known as vitamin P, is a bioflavonoid with antitumoral and antioxidant activities. The reno-protective actions of rutin in obstructive nephropathy are due to its anti-inflammatory actions and its ability to inhibit the TGF-β1/Smad3 signaling. At the same time, a report demonstrated that rutin operates as a powerful antioxidant in the defense from kidney injury caused by Cd [133].

A study demonstrated the action of rutin on CFZ-caused kidney damage through the reduction in inflammation and oxidative stress [134]. CFZ administration remarkably reduced the number of antioxidant enzymes; while the employment of rutin and olmesartan upturned their concentrations toward normality. Moreover, the concentrations of caspase-3 were decreased by rutin administration, and the histopathological assessment confirmed that rutin has protective action against CFZ-caused nephrotoxicity [134]. Different experiments demonstrated that rutin administration was also able to decrease the CFZ-provoked modification in cardiac enzymes, rutin treatment prevented the activation of NF-kB by increasing the expression of the inhibitory protein IkB alpha, and reduced the action of CFZ in oxidant–antioxidant systems [135].

Other substances might be helpful to prevent the CFZ toxic effects on cardiac tissues, and a study evaluated the possible protective action of apremilast (AP), a phosphodiesterase 4 (PDE4) inhibitor [136]. The administration of CFZ provoked a significant increase in cardiac enzymes, which were normalized by AP administration. Furthermore, AP administration reversed the increase in heart MDA amounts and the reduction in cardiac GSH concentrations induced by CFZ treatment [136].

Appropriate modulation of oxidative stress could also be helpful to reduce the onset of side effects due to drugs used for treating complications of MM such as bone disease. Bisphosphonates (BPs) are commonly utilized medicines that seem to have an additional impact on MM progression. BPs are potent inhibitors of osteoclast-caused bone resorption used to treat MM patients with osteolytic lesions. Osteonecrosis of the jaw (ONJ) is a rare BPs-caused adverse event of these drugs. The precise mechanisms of ONJ are not recognized, and its pathogenesis is founded on numerous suppositions, such as a reduction in bone remodeling or suppressive action on angiogenetic dynamics [137,138].

Some research has stated oxidative damage due to BPs in different normal and tumoral cells or tissues [139]. Karabulut et al. evaluated oxidative stress modifications after BP administration assessing GSH and MDA concentrations in rabbit liver cells [140]. They reported more significant MDA amounts and smaller GSH concentrations in animals treated with zoledronic acid. These data propose that similar oxidative stress modifications might be present in subjects after BP treatment, particularly in patients with inflammatory conditions, such as in ONJ.

Recently, a study reported that BPs stimulate fibroblasts isolated from the oral cavity to produce ROS. The following ROS-caused suppression of fibroblast proliferation and migration delayed wound healing, participating in ONJ occurrence [141].

Furthermore, in a different study, mean serum and saliva concentrations of MDA, glutathione disulfide (GSSG), and the GSSG/GSH ratio were remarkably greater in ONJ subjects than in healthy controls, and regression analysis demonstrated that the GSSG/GSH ratio was a critical element predicting the occurrence of ONJ [142].

The critical question is how to cope with multiple myeloma subjects treated with probably toxic drugs and if it is possible to reduce or prevent chemotherapy-caused side effects without diminishing the potentially life-saving treatment. Currently, guidelines do not recommend any agent to avoid collateral drug effects, and the collaboration between preclinical and clinical scientists is indispensable to transform an enhanced comprehension of the mechanisms of drug toxicities into efficacious strategies. Harnessing cellular redox parameters might be a productive strategy to prevent treatment side effects.

## 7. Conclusions

In conclusion, an alteration of intracellular oxidative stress is often associated with malignant transformation of plasma cells owed to both oncogene stimulation and augmented metabolism in clonal cells. Consequently, plasma cells present higher amounts of ROS and smaller concentrations of antioxidant molecules with respect to their normal counterparts. Deranged generation of ROS induces oxidative stress, which could be harmful to the cells, and additional stimulation of oxidative stress can be a practical approach to treat MM. Actually, combined administration of anti-myeloma drugs with other ROS-stimulating compounds confirmed encouraging results, and a therapeutic strategy founded on the relevant diversity existing in cancer and normal cell oxidative metabolism opens novel possibilities for MM patients and combining oxidative stressors with traditional chemotherapy may be conducted to implement more effective treatment strategies.

However, more studies are needed to understand the close relationships between oxidative stress and myelomatous disease. All to be discovered are, for example, the therapeutic implications that could arise from the study of the immunoproteasome. In fact, in addition to the constitutive proteasome [143,144], which is expressed in all cells and tissues, higher organisms such as vertebrates possess two immune-type proteasomes, the thymoproteasome and the immunoproteasome [26,145]. The latter is a specific proteasome isoform induced by interferons. Recent experiments demonstrate that immunoproteasome is essential for efficiently eliminating nascent oxidant damaged proteins and for protecting toxic aggregate formation under inflammatory conditions. This function apparently cannot be sufficiently fulfilled by 26S s-proteasomes. At the same time, immunoproteasome also permits the timely availability of regulators modulating cytokine generation and cellular growth [26,145].

Recent studies demonstrated that the immunoproteasome has a preservative role during oxidative stress and is upregulated in many pathological disorders, including cancer, inflammatory, and autoimmune diseases. Consequently, immunoproteasome-selective inhibitors are currently the focus of anticancer drug design [26]. Identifying a synergistic action of oxidative stress modifiers with immunoproteasome inhibitors could open enormous research spaces for the treatment of relapsing MM.

Another stimulating field of study could be constituted by deepening the relationship between daratumumab (DARA), an essential drug for the treatment of myeloma and oxidative stress, and their effects on MM cells. A study employed the CRISPR/Cas9 system to delete CD38 (CD38KO) in ex vivo expanded peripheral blood NK cells. These CD38KO NK cells were utterly resistant to DARA-induced fratricide, showed superior persistence in immune-deficient mice pretreated with DARA, and enhanced ADCC activity against CD38-expressing MM cell lines and primary MM cells. Transcriptomic and cellular metabolic analysis demonstrated that CD38KO NK cells have unique metabolic reprogramming with a higher mitochondrial respiratory capacity [146]. A better understanding of the effects of DARA on oxidative metabolism could allow for more effective and safer use of this drug.

Finally, besides offering more significant curative benefits, such new therapeutical strategies could also specifically reduce the occurrence of chemoresistance in MM cells, which is the principal cause of treatment failure in most MM patients. On the other hand, manipulating oxidative stress could be helpful to reduce the side effects and toxicity of anti-myeloma therapy.

## Figures and Tables

**Table 1:** Alteration of the redox mechanisms found in patients with multiple myeloma.

Parameters	Material	Status	Ref.
Vitamin C, vitamin E, glutathione peroxidase, superoxide dismutase catalase	Serum	Reduced	[11,12,13,14,15]
Advanced oxidation protein products, malondialdehyde, markers of fatty acid peroxidation	Serum	Augmented	[11,12,13,14,15]
Paraoxonase, arylesterase	Serum	Reduced	[16,17]
Nicotinamide adenine dinucleotide	Peripheral blood mononuclear cells	Reduced	[18]
Hydrogen peroxide	Peripheral blood mononuclear cells	Augmented	[18]

**Table 2:** Effects of substances of natural origin in the modulation of oxidative stress in multiple myeloma, mechanisms of action, targets, and synergistic effects with proteasome inhibitors.

Molecules	Effects on Oxidative Stress	Mechanisms of Action	Ref.
Curcumin and Carfilzomib	Augmented ROS concentrations	They stimulated the p53/p21 axis and G0/G1 cell cycle arrest, and curcumin augmented the CFZ proapoptotic effect	[109]
Sanguinarine and Bortezomib	Mitochondrial membrane potential loss	They increased apoptosis and caused inhibition of the STAT3 pathway	[110]
*Salvia miltiorrhiza*	Augmented ROS production and ER stress	Augmented apoptosis and increased miRNA-216b expression	[114]
Caffeic acid phenethyl ester	Stimulation of oxidative stress-response genes (heme oxigenase-1). Reduced intracellular antioxidants	Reduced MM cells proliferation	[115]
Resveratrol and Carfilzomib	Augmented ROS production. Delivery of second mitochondria-derived activator of caspase	Augmented apoptosis	[116]
Formononetin	Augmented generation of ROS for a GSH/GSSG disequilibrium	Decrease in kinases such as c-Src, JAK1, and JAK2	[117]
Plitidepsin	Augmented oxidative and ER stress, JNK1, and eIF2α phosphorylation	Increased apoptosis	[118]
Curcusone D and Bortezomib	Augmented ROS generation	Ubiquitin–proteasome pathway inhibition	[119]
